# Retraction: Enhanced IMP3 Expression Activates NF-кB Pathway and Promotes Renal Cell Carcinoma Progression

**DOI:** 10.1371/journal.pone.0301575

**Published:** 2024-04-02

**Authors:** The PLOS ONE Editors

This article [1] was identified as one of a group of articles connected by concerns about image reuse [2–13].

The concerns about this article [1] affect Figs 2, 3, and 4. Specifically,

The following panels appear similar:

- Fig 2A IMP3 panel of [1] and Fig 2D ACHN siRNA panel of [2].
  ○ Fig 2B Consh panel of [1], Fig 2D 786–0 Control panel of [2], and Fig 2D sh-NC Caki-2 panel of [3].
  ○ Fig 2B IMP3sh panel of [1], Fig 2D 786–0 siRNA panel of [2], and Fig 2F sh-ROR Caki-1 panel of [3].
  ○ Fig 2C IMP3 panel of [1] and Fig 2E ACHN siRNA panel of [2]
  ○ Fig 2D Consh panel of [1], Fig 2D ACHN Control panel of [2], Fig 2E ACHN Control panel of [2], and Fig 2F sh-NC Caki-1 panel of [3].
  ○ Fig 2D IMP3sh panel of [1], Fig 2E 786–0 siRNA panel of [2], and Fig 2F sh-NC Caki-2 panel of [3].
  ○ Fig 2E ConsiRNA panel of [1] and Fig 2D sh-NC Caki-1 panel of [3].
  ○ Fig 2E IMP3siRNA panel of [1], Fig 2E 786–0 Control panel of [2], and Fig 2D sh-ROR Caki-1 panel of [3].
  ○ Fig 4A FLAG+BAY 11–7082 panel of [1] and Fig 2F sh-ROR Caki-2 panel of [3].
  ○ Fig 4A FLAG-IMP3 panel of [1] and Fig 2D shROR Caki-2 panel of [3].
- In the Fig 3H Actin panel there appears to be a vertical irregularity suggestive of a splice line between lanes 2–3.

The corresponding authors stated they were unaware of the panel duplications with [2 and 3]. They provided data files to support the results presented in this article but the files did not resolve the concerns.

The *PLOS ONE* Editors retract this article [1] due to the above image concerns that call into question the reliability of the reported results.

ML, HH, MZ, HZ, and XP did not agree with the retraction and stand by the article’s findings. JZ, YY, XW, LG, HA, and PE either did not respond directly or could not be reached.
